# Harnessing miRNA-Containing Extracellular Vesicles from Mesenchymal Stromal Cell-Derived Extracellular Vesicles for Regeneration of Bone Defects: A Narrative Review of Mechanisms, Biomaterials, and Clinical Translation

**DOI:** 10.3390/cancers17152438

**Published:** 2025-07-23

**Authors:** Kashia Goto, Daisuke Watanabe, Kazuki Yanagida, Tatsuya Takagi, Akio Mizushima

**Affiliations:** 1 Department of Palliative Medicine, Juntendo University Graduate School of Medicine, Tokyo 113-8421, Japan; k.goto.ew@juntendo.ac.jp (K.G.); ttatsuya@juntendo.ac.jp (T.T.); akiom@juntendo.ac.jp (A.M.); 2 Department of Urology, Koto Hospital, Tokyo 135-0034, Japan; k.yanagida.sr@juntendo.ac.jp; 3 Department of Molecular and Cellular Therapeutics, Juntendo University Graduate School of Medicine, Tokyo 113-8421, Japan

**Keywords:** bone defect, bone regeneration, mesenchymal stromal cell-derived extracellular vesicles, MSC-EVs, microRNA

## Abstract

We present a narrative review focusing on the therapeutic potential of mesenchymal stromal cell-derived extracellular vesicles (MSC-EVs) in regenerating bone defects, particularly those resulting from surgical treatment of malignant bone and soft tissue tumors. These large bone defects pose significant challenges for reconstruction and functional recovery, highlighting the need for innovative regenerative strategies. **Background**: MSCs, which can differentiate into various cell types, are known for their immunosuppressive properties and ability to promote tissue repair. MSC-EVs, rich in bioactive molecules like microRNAs and proteins, play a crucial role in bone regeneration by mediating intercellular communication and modulating inflammation. **Methods**: This narrative review compiles data from various studies, including systematic reviews and individual research, focusing on the application of MSC-EVs in bone defect treatment. It examines the characteristics, mechanisms of action, and therapeutic effects of MSC-EVs, as well as the microRNAs involved in bone regeneration. **Results**: The findings indicate that MSC-EVs can enhance both osteogenesis and angiogenesis, highlighting their potential as promising candidates for clinical applications in bone defects. However, many mechanisms remain unclear; therefore, further investigation is needed. **Conclusions**: The review emphasizes the potential of MSC-EVs in improving patient outcomes for severe bone defects. It also highlights future challenges, including formulation, standardization, safety, and delivery methods, particularly in conjunction with biomaterials. Overall, MSC-EVs represent a significant advancement in regenerative medicine for bone defects.

## Simple Summary

This narrative review focuses on the potential of mesenchymal stromal cell-derived extracellular vesicles (MSC-EVs) in addressing bone defects that arise following surgical treatment of malignant bone and soft tissue tumors. MSC-EVs are recognized for their ability to deliver bioactive molecules, such as microRNAs and proteins, which promote bone formation and blood vessel growth. Research on the clinical application of MSC-EVs for bone repair is advancing rapidly; however, many of their mechanisms remain unclear, and further investigation is needed. MSC-EVs can be sourced from various tissues, including the bone marrow, adipose tissue, and dental pulp, each of which offers unique therapeutic benefits. This review discusses the characteristics, mechanisms of action, and effects of MSC-EVs, as well as the microRNAs involved in bone regeneration. It also highlights future challenges in clinical applications, such as formulation, standardization, safety, and delivery methods, particularly in combination with biomaterials. Overall, MSC-EVs represent a promising strategy for enhancing bone regeneration and improving patient outcomes.

## 1. Introduction

The surgical treatment of bone and soft tissue tumors, including primary bone sarcomas and aggressive soft tissue malignancies with bone infiltration, often requires wide resection of the affected bone [1,2]. Such procedures frequently result in large segmental bone defects that present significant challenges for reconstruction. In addition, cancers such as prostate cancer are known for their high propensity to metastasize to the bone, most commonly to the spine, but also frequently to the pelvis and femur. In selected patients with solitary or oligometastatic lesions, palliative surgical resection of metastatic bone sites, particularly in weight-bearing bones such as the femur, combined with fixation plates or prosthetic reconstruction, is sometimes performed to relieve pain, preserve ambulation, and maintain activities of daily living (ADLs) [3]. These diverse clinical scenarios share a common therapeutic goal: effective reconstruction of bone defects to restore function and improve quality of life. Nonetheless, existing reconstructive options are limited by donor site morbidity, risk of infection, mechanical failure, and suboptimal biological integration.

Bone regeneration remains a major challenge in regenerative medicine, particularly in conditions such as periodontitis, osteoporosis, and traumatic injuries. Conventional approaches often involve invasive surgical techniques or the use of biomaterials, which may not fully support the biological processes essential for effective healing [4].

Mesenchymal stromal cell-derived extracellular vesicles (MSC-EVs) are increasingly recognized as promising cell-free therapies for bone regeneration, particularly in periodontal bone defects. MSC-EVs are rich in bioactive molecules, such as microRNAs, messenger RNAs (mRNAs), proteins, and cytokines, which promote various regenerative processes. MSC-EVs have been shown to enhance osteogenic differentiation and angiogenesis, thereby promoting overall tissue regeneration.

Future research should focus on optimizing the production, isolation, and characterization of MSC-EVs to enhance their therapeutic efficacy. There is growing interest in engineering approaches to improve the delivery and functionality of MSC-EVs and in studying the specific bioactive molecules responsible for their regenerative effects. Understanding the molecular mechanisms underlying MSC-EV action is crucial for advancing their clinical application in bone regeneration and addressing challenges such as variability in trial results and potential side effects [5,6]. One potential side effect is the exacerbation of malignant tumors. Extracellular vesicles derived from various sources can exacerbate malignancy through multiple interconnected mechanisms, including promoting tumor cell proliferation, modulating the tumor microenvironment (TME), inducing epigenetic alterations, enhancing angiogenesis, and enabling immune evasion. This complex interplay underscores the importance of further research to develop effective cancer therapies. However, MSC-EVs are considered a versatile and effective strategy for promoting bone regeneration, with significant potential to improve the outcomes of patients with severe bone defects.

This narrative review aims to provide an overview of the promising potential of MSC-EVs in the treatment of bone defects, highlighting their characteristics, mechanisms of action, therapeutic effects, and future clinical challenges and perspectives as current topics. We hope this review serves as a foundation for future research that advances the clinical application of MSC-EVs in bone defects.

## 2. Methods

Using information from reviews, systematic reviews, and individual studies on the application of MSC-EVs for bone defects, we compiled data on MSC-EVs in the context of bone defect treatment. This narrative review focuses on the latest research trends and challenges in the clinical application of MSC-derived exosomes for bone defects, including formulation, preservation, administration methods, and progress in clinical trials. Furthermore, we summarized the characteristics and mechanisms of MSC-EVs in bone regeneration, the microRNAs encapsulated within EVs involved in bone regeneration, and the materials used for EV delivery and bone regeneration. Specifically, we organized information using search terms such as “Bone defect”, “MSC-EV”, “Mechanism”, “Effect”, “ADSC-EV”, “BMSC-EV”, “DPSC-EV”, “EV-Encapsulated microRNA”, “Biomaterial”, “EV Transport”, “Clinical Trials”, “Challenges”, and “Perspectives”. We searched PubMed for full-text reviews, systematic reviews, and individual studies published up to May 2025 that involved microRNAs encapsulated within EVs related to bone regeneration. In this review, we summarize recent advances in MSC-derived exosome therapies for bone defect repair.

## 3. Characteristics, Mechanisms of Action, Effects, and Future Research Directions of MSC-Derived Exosomes in Bone Defects

Mesenchymal stromal cells (MSCs) are a type of multipotent stem cell that can differentiate into various cell types, including bone, cartilage, and fat cells. They are primarily found in the bone marrow but can also be isolated from other tissues such as adipose tissue and umbilical cord blood [7]. MSCs are known for their immunosuppressive properties, which means they can modulate immune responses, reduce inflammation, and promote tissue repair and regeneration [7].

Extracellular vesicles (EVs), especially those derived from MSCs, play an important role in systemic and interorgan effects on bone regeneration. Their ability to mediate communication, modulate inflammation, and influence immune responses positions them as promising therapeutics in regenerative medicine, and the exploration of different cell sources for EV production further enhances their potential application in the treatment of bone-related diseases.

### 3.1. Adipose-Derived Stem Cell Extracellular Vesicles (ADSC-EVs)

Adipose-derived stem cell extracellular vesicles (ADSC-EVs) are small membrane-bound vesicles (30–150 nm) that play a crucial role in intercellular communication and demonstrate significant therapeutic potential in regenerative medicine. ADSC-EVs are rich in bioactive molecules, including proteins, lipids, and various RNAs and microRNAs, which regulate cellular functions such as inflammation, apoptosis, and tissue regeneration. ADSC-EVs promote angiogenesis, enhance cell survival, and accelerate wound healing, particularly in diabetic and osteoporotic wound models [8,9]. Their mechanisms of action primarily involve the activation of signaling pathways such as PI3K/Akt and STAT3, which are essential for promoting cell proliferation, migration, and differentiation [10,11].

Future research is expected to focus on optimizing the isolation and characterization of ADSC-EVs, understanding their molecular mechanisms in various therapeutic contexts, and evaluating their safety profiles for clinical applications. Studies investigating the specific microRNA content of ADSC-EVs are increasing, with findings suggesting their role in regulating macrophage polarization and promoting tissue repair [9,12]. Additionally, the potential of ADSC-EVs as a cell-free therapy is being explored, particularly for chronic diseases and conditions in which traditional stem cell therapies face challenges such as immunogenicity and ethical concerns [8,13]. The therapeutic application of ADSC-EVs is anticipated to advance regenerative medicine and improve patient outcomes in various clinical settings.

### 3.2. Bone Marrow-Derived Mesenchymal Stromal Cell Extracellular Vesicles (BMSC-EVs)

Bone marrow-derived mesenchymal stromal cell extracellular vesicles (BMSC-EVs) have shown significant potential in promoting bone regeneration and treating conditions such as osteoporosis. BMSC-EVs are typically characterized by a lipid bilayer structure with a diameter of 30–200 nm, containing various bioactive molecules including proteins, lipids, and RNAs, which facilitate intercellular communication and influence the behavior of recipient cells [14,15]. Mechanistically, BMSC-EVs promote osteogenesis by activating key signaling pathways such as Wnt/β-catenin and BMP-2/Smad1/RUNX2, which are crucial for osteoblast differentiation and function. For instance, Xuepeng Wang et al. demonstrated that BMSC-EVs stabilize YAP1 protein through ubiquitin-specific peptidase 7 (USP7)-mediated deubiquitination, thereby enhancing bone formation in osteoporosis models [15]. Ba Huang et al. reported that BMSC-EVs modified with GPNMB significantly improve trabecular bone regeneration in ovariectomized rat models by stimulating osteogenic differentiation via the Wnt/β-catenin pathway [14].

Future research is expected to focus on optimizing the therapeutic efficacy of genetically engineered BMSC-EVs to enhance their osteogenic potential and explore their clinical applications for bone repair. Studies on specific cargoes of BMSC-EVs, such as microRNAs and proteins, are essential for understanding their mechanisms of action and improving their therapeutic applications [13,16]. Comparative studies of EVs derived from various sources, such as adipose tissue and umbilical cord, may provide insights into their unique properties and functions, potentially leading to personalized regenerative therapies [17,18]. The exploration of BMSC-EVs represents a promising frontier in regenerative medicine, particularly for promoting bone healing and addressing osteoporotic conditions in the future.

### 3.3. Dental Pulp Stem Cell-Derived Extracellular Vesicles (DPSC-EVs)

Dental pulp stem cell-derived extracellular vesicles (DPSC-EVs) have significant potential for bone regeneration, particularly in maxillofacial defects. DPSC-EVs are characterized by their ability to promote osteogenic differentiation in MSCs via paracrine signaling mechanisms. Studies have demonstrated that DPSC-EVs enhance the expression of osteogenic markers, such as RUNX2, alkaline phosphatase (ALP), and osteocalcin (OCN), in recipient cells, comparable to the effects of established osteoinductive factors, such as BMP-2 [19,20]. The mechanism of action involves the activation of the MAPK signaling pathway, particularly through the ERK and JNK pathways, which are crucial for mediating the osteogenic effects of DPSC-EVs [3]. The efficacy of DPSC-EVs in promoting bone regeneration is further supported by in vivo studies, which show enhanced new bone formation in rat models of critical-sized defects treated with DPSC-EVs in combination with various scaffolds [20].

Future research is likely to focus on optimizing the therapeutic applications of DPSC-EVs, including the identification of specific proteins and microRNAs within these vesicles that contribute to their regenerative properties. Additionally, studies should explore the synergistic effects of DPSC-EVs with different biomaterials to enhance their osteogenic potential and investigate their role in modulating immune responses during tissue repair [20,21]. The development of standardized protocols for the isolation and characterization of DPSC-EVs is critical for advancing their clinical applications in regenerative medicine [19]. Overall, DPSC-EVs represent a promising avenue for cell-free therapies in bone regeneration, with the potential to overcome some of the limitations associated with traditional stem cell therapies.

### 3.4. Human Umbilical Cord Mesenchymal Stem Cell-Derived Extracellular Vesicles (HucMSC-EVs)

Human umbilical cord mesenchymal stem cell-derived extracellular vesicles (HucMSC-EVs) are nanoscale vesicles ranging from 40 to 160 nm in diameter, characterized by their rich content of bioactive molecules, including proteins, lipids, and microRNAs [22,23]. The EVs are known for their high biocompatibility and low immunogenicity, making them attractive for therapeutic applications [22,23]. They are identified by specific surface markers such as CD9, CD63, and CD81, which indicate their potential for effective cellular interactions [22,23]. HucMSC-EVs primarily function by modulating various biological processes, including enhancing osteogenic differentiation and promoting angiogenesis through the activation of critical signaling pathways such as Wnt and Hippo [22,23]. Additionally, they exhibit immunomodulatory effects by promoting the polarization of macrophages towards an anti-inflammatory phenotype, which is beneficial for tissue repair [22,23]. Current research trends focus on optimizing the therapeutic potential of HucMSC-EVs through innovative delivery systems and biomaterials to enhance their bioavailability and efficacy at target sites [22,23]. For instance, encapsulating HucMSC-EVs in biodegradable polymers has been explored to achieve sustained release [22,23]. Studies are also investigating the specific microRNAs and proteins within these exosomes that mediate their effects, aiming to develop targeted therapies for conditions such as bone defects and inflammatory diseases [22,23]. Overall, the promising results from ongoing research highlight the potential of HucMSC-EVs as a novel therapeutic approach in regenerative medicine, particularly in enhancing bone healing and reducing inflammation [22,23].

### 3.5. Other EVs That May Be Involved in Bone Regeneration

Potential involvement in bone regeneration through small molecules, direct cell migration, or EV secretion, including stem cell types from other organs, and mechanisms of action are summarized.

Mesoangioblasts (MABs), multipotent skeletal muscle progenitor cells, can differentiate into osteoblasts in response to DNA damage [24]. This process is induced by Idoxuridine (IdU), which activates the DNA Damage Response, involving ATM kinase and p38 MAPK pathways. These pathways lead to the phosphorylation of RUNX2, a key regulator of osteogenic differentiation. RUNX2 promotes the expression of osteogenic markers, facilitating bone formation. This mechanism highlights the role of DNA damage in bone regeneration and conditions like heterotopic ossification.

Muscle stem cells, known as satellite cells, have limited involvement in ectopic ossification (bone formation in non-skeletal tissues). Vitali Y Lounev et.al. showed that these cells contribute minimally to heterotopic ossification induced by BMP2 [25]. Instead, the inflammatory environment and BMP signaling are more critical, with endothelial cells expressing the Tie2 marker being the major contributors. These endothelial progenitor cells are involved in all stages of ectopic bone formation, unlike muscle-derived satellite cells, which primarily aid in muscle repair and regeneration.

Skeletal muscle secretes extracellular vesicles (Mu-EVs) that travel through the bloodstream to bone tissue, influencing bone health. Mu-EVs contain bioactive factors that promote the osteogenic differentiation of bone marrow mesenchymal stem/stromal cells (BMSCs) and enhance glycolysis, stimulating bone formation. The secretion and bioactivity of Mu-EVs are linked to muscle activity, with increased activity enhancing bone health and muscle atrophy reducing it. Mu-EVs show potential as a therapeutic strategy for bone degenerative diseases associated with muscle atrophy [26].

Extracellular vesicles (EVs) play a crucial role in communication between the liver and other organs, including bone tissue, which is vital for maintaining physiological balance and responding to diseases like hepatic osteodystrophy. Liver-derived EVs can regulate the activity of osteoblasts and osteoclasts, impacting bone density and health in liver disease patients. The pathways through which EVs affect bone regeneration involve signaling molecules such as hormones and cytokines, which modulate the balance between bone formation and resorption.

### 3.6. Systemic and Cross-Organ Effects of EVs

EVs have various effects on cells, tissues, and organs. Among these actions, anti-inflammatory, immunomodulatory, and tissue repair actions are expected to be utilized as therapeutic agents for specific diseases. MSC-derived EVs are of particular interest [27]. For example, clinical studies of umbilical cord-derived MSCs added to the inner ear to reduce inflammation as an adverse reaction to cochlear implantation have shown improved speech perception [28]. ADSC-EVs are expected to reduce the size of amyloid-beta plaques and improve cognitive impairment in the treatment of Alzheimer’s disease, in addition to rheumatoid arthritis and osteoarthritis through anti-inflammatory effects. Robert Mitchell et.al implied that extracellular vesicles and soluble molecules within the ADSC secretome act in a synergistic manner to promote muscle generation [29].

These suggest that EVs may affect multiple organ systems and may be beneficial in the treatment of diseases that indirectly affect bone health, such as systemic inflammation and metabolic disorders.

## 4. EV-Encapsulated microRNAs Involved in Bone Regeneration and Their Mechanisms of Action

Extracellular vesicles derived from MSCs play a crucial role in bone regeneration by encapsulating various microRNAs that regulate key biological processes. We summarized EV-encapsulated microRNAs involved in bone regeneration and their mechanisms of action [30,31,32,33,34,35,36,37,38] (see Table 1). These microRNAs promote osteogenic differentiation, enhance cell proliferation, and facilitate overall healing. For example, miR-100-5p and miR-21-5p from adipose-derived MSCs enhance osteogenic differentiation by modulating signaling pathways related to bone formation. miR-21-5p is particularly associated with cell survival and proliferation, essential for effective bone regeneration [26].

Bone marrow MSCs also contribute significantly to bone healing through their EV-encapsulated microRNAs, such as miR-27a and miR-335-5p, which promote osteogenic differentiation and enhance bone healing by targeting genes involved in bone metabolism [30,32,33,36]. These microRNAs activate pathways like Wnt/β-catenin, crucial for bone formation and repair. Additionally, miR-92a influences angiogenesis, supporting the vascularization necessary for effective bone healing. The interplay of different microRNAs encapsulated in EVs from various MSC sources collectively impacts bone regeneration, highlighting their potential in developing targeted therapies for improving bone healing and addressing conditions like osteoporosis [30,31,32,33,34,35,36,37,38] (Table 1).

On the other hand, Virinder Kaur Sarhadi et al. [39] reported that EVs derived from BMSC-EVs and ADSC-EVs have been shown to promote tumor aggressiveness in OS. A key component of these EVs is microRNA-208a (miR-208a), which significantly enhances the malignancy of tumor cells. BMSC-EVs carry miR-208a, which promotes OS cell migration and invasion by enhancing the expression of genes associated with tumor progression. Specifically, miR-208a activates the JAK2/STAT3 signaling pathway, which is crucial for cancer cell proliferation and survival. The presence of miR-208a in EVs from BMSC-EVs also leads to increased migration and invasion of OS cells through the upregulation of matrix metalloproteinases. These enzymes degrade extracellular matrix components, facilitating the invasion of cancer cells into surrounding tissues. Through these mechanisms, miR-208a in BMSC-EVs and ADSC-EVs plays a pivotal role in promoting tumor aggressiveness, making it a potential target for therapeutic intervention in OS [39].

**Table 1:** EV-Encapsulated microRNAs and Their Mechanisms in Bone Regeneration.

EV-Encapsulated microRNA	Mechanism of Action	EV Origin MSC/Source	Refs.
miR-148a-3p	Influences osteogenic differentiation and regulates osteoblast activity. Targets Smad ubiquitination regulatory factor 1 (SMURF1) in the SMAD signaling pathway, preventing the degradation of SMAD7. Promotes the expression of the anti-apoptotic protein BCL2, enhancing the viability and osteogenic potential of BMSCs. Promotes osteoblast differentiation by targeting specific genes involved in the osteogenic pathway. Enhances the expression of osteogenic markers, contributing to bone formation.	ADSCs BMSC-EVs ADSC-EVs	[31,36,38]
let-7 g-5p	Involved in the regulation of osteogenic differentiation and apoptosis in osteoblasts	ADSCs	[36]
let-7a	Involved in the regulation of osteogenic differentiation and matrix mineralization.	hASCs-EVs	[30,32,33]
let-7i-5p	Promotes osteogenic differentiation of MSCs by targeting negative regulators of osteogenesis	ADSC-EVs	[31]
miR-100	Promotes osteogenic differentiation by enhancing the expression of osteogenic markers.	MSCs-EVs	[36]
miR-100-5p	Involved in the regulation of signaling pathways that facilitate bone formation.	ADSCs	[33]
miR-122-5p	Inhibits Sprouty2 (SPRY2), a receptor tyrosine kinase inhibitor in the MAPK pathway, thereby increasing MAPK pathway activity. Enhances the expression of RUNX2 and type I collagen (COL-I), promoting osteoblast proliferation	BMSC-EVs	[38]
miR-148a	Influences osteogenic differentiation and regulates osteoblast activity, contributing to bone formation.	MSCs-EVs	[33]
miR-151a-3p	Regulates osteoblast activity, enhancing their proliferation and function	ADSC-EVs	[31]
miR-196a	Regulates osteoblastic differentiation and promotes the expression of osteogenic genes.	hBMSC-EVs	[30,32]
miR-199b-5p	Enhances osteogenic differentiation and promotes bone regeneration by targeting specific genes involved in bone metabolism	BM-MSCs	[36]
miR-200b	Influences osteogenic differentiation and may play a role in the regulation of signaling pathways involved in bone regeneration	BM-MSCs	[36]
miR-206	Enhances osteogenic differentiation and promotes the expression of key osteogenic markers.	hBMSC-EVs	[30,32]
miR-21	Enhances osteogenic differentiation and promotes bone healing by targeting genes involved in bone metabolism. Activates the PI3K/Akt signaling pathway, promoting cell survival and proliferation	MSCs-EVs HucMSCs-EVs	[31,33,38]
miR-210	Inhibits excessive activation of the PI3K/AKT/mTOR pathway, reducing endothelial cell apoptosis	BMSC-EVs	[38]
miR-21-5p	Enhances osteogenic differentiation and promotes bone healing by targeting specific genes involved in bone metabolism	ADSCs	[36]
miR-217	Involved in the regulation of osteogenic differentiation and may influence the expression of osteogenic markers	BM-MSCs MSCs-EVs	[36] [33]
miR-218	Promotes bone regeneration by increasing the expression of osteogenic markers such as RUNX2 and ALP.	hASCs-EVs	[30,32]
miR-22-3p	Inhibits the MYC/PI3K/AKT signaling pathway by targeting the fat mass and obesity-associated protein.	BM-MSCs	[30,35]
miR-24 miR-24-3p	Regulates osteogenic differentiation and affects the expression of key osteogenic markers.	MSCs-EVs	[33,36]
miR-26a	Enhances osteogenic differentiation and promotes bone formation by targeting specific genes.	BM-MSCs MSCs-EVs	[33,36]
miR-27a	Promotes osteogenic differentiation and enhances bone regeneration by modulating target genes involved in bone metabolism. Promotes osteogenic differentiation and enhances the expression of osteogenic markers such as osteocalcin (OCN) and osteopontin (OPN).	hBMSC-EVs BM-MSCs	[30,32,33,36]
miR-29b-3p	Promotes angiogenesis during fracture healing by being encapsulated in EVs and taken up by endothelial cells (e.g., HUVECs). Suppresses the expression of PTEN, a negative regulator of the PI3K/AKT signaling pathway, leading to enhanced cell proliferation and migration.	BM-MSCs	[30,34]
miR-3084-3p	Upregulates RUNX2 and ALP, which are critical for osteogenic differentiation and matrix mineralization. Activates the Wnt/β-catenin signaling pathway, promoting bone formation.	BM-MSC-EVs	[32]
miR-335	Promotes osteogenic differentiation and enhances bone healing by modulating target genes involved in bone metabolism. Promotes osteoblast differentiation and bone fracture recovery by targeting VapB. Activates the Wnt/β-catenin signaling pathway, which is crucial for bone formation and repair.	MSCs-EVs BM-MSCs	[33,40]
miR-335-5p	Promotes osteogenic differentiation and enhances bone healing by modulating target genes.	BM-MSCs	[36]
miR-34a	Enhances bone regeneration by increasing the expression of RUNX2, ALP, and collagen type I (COL1A1).	hADSCs-EVs	[30,33]
miR-375	Improves osteogenic differentiation by inhibiting IGFBP3, which is involved in regulating growth factors.	hASCs-EVs	[30,33]
miR-378	Suppresses the expression of the Hh pathway inhibitor suppressor of fused homolog (Sufu), promoting the production of VEGF and angiopoietin-1 (ANG-1).	ADSCs	[38]
miR-5100	Contributes to the upregulation of osteogenic markers, promoting bone regeneration.	BM-MSC-EVs	[32]
miR-677-3p	Involved in the regulation of osteogenic differentiation through the modulation of key osteogenic genes.	BM-MSC-EVs	[32]
miR-680	Contributes to the upregulation of RUNX2 and ALP, facilitating osteogenic differentiation.	BM-MSC-EVs	[32]
miR-9	Regulates osteogenic differentiation and may affect the expression of osteogenic markers.	BM-MSCs	[36]
miR-92a	Involved in the regulation of osteogenic differentiation and may influence angiogenesis.	BM-MSCs	[36]

miR, microRNA; ADSC, adipose-derived mesenchymal stem cell; hASCs-EVs, human adipose-derived stem cell-derived extracellular vesicles; EVs, extracellular vesicles; MSCs, mesenchymal stem cells; hBMSC-EVs, human bone marrow stem cell-derived extracellular vesicles; BM-MSCs, bone marrow-derived mesenchymal stem cells; HucMSCs-EVs, human umbilical cord MSC-derived extracellular vesicles.

## 5. Materials and Their Characteristics for EV Transport and Bone Regeneration

Extracellular vesicles are increasingly recognized for their potential in bone regeneration, and the materials used for their transport play a crucial role in enhancing their therapeutic efficacy. Various biomaterials, including biodegradable polymers, scaffolds, hydrogels, and bioactive coatings, have been developed to optimize the delivery and retention of EVs at bone defect sites. Each material type possesses unique characteristics that contribute to its effectiveness in promoting bone healing and regeneration. The data related to materials and their type, characteristics for EV transport, and bone regeneration are shown in Table 2. Biodegradable polymers such as poly lactic-co-glycolic acid (PLGA) and poly ethylene glycol (PEG) are engineered to facilitate controlled release of EVs over a tunable time scale [41]. These polymers are biocompatible, allowing for integration within three-dimensional tissue engineering scaffolds, which simplifies surgical insertion. The degradation of PLGA into safe byproducts makes it particularly suitable for medical applications, ensuring that the material does not pose long-term risks to the patient while supporting the gradual release of therapeutic agents. Several types of scaffolds, including ceramic, polymer, and composite scaffolds, provide structural support for cell attachment and proliferation, which are essential for bone healing. For instance, ceramic scaffolds are known for their high biocompatibility and bioactivity, supporting bone mineralization and allowing for nutrient exchange due to their porous nature [42]. Polymer scaffolds, on the other hand, can be tailored for specific mechanical properties and controlled degradation, ensuring that they match the requirements of the regenerating bone tissue [42]. Additionally, composite scaffolds combine the beneficial properties of both ceramics and polymers, enhancing mechanical strength and bioactivity [42]. Hydrogels, particularly those based on hyaluronic acid, offer a hydrated environment that mimics natural tissue, promoting cell survival and function [42]. Their high-water content and flexibility allow them to replicate the extracellular matrix, providing structural support for cells. Hydrogels can also facilitate enhanced cell communication and interaction with EVs, potentially improving the regenerative process. Moreover, they are capable of controlled release of EVs, which enhances the therapeutic effects over time. Collagen sponges are natural biomaterials that promote biocompatibility and integration with host tissues [43]. Their sponge-like structure allows for cell infiltration and nutrient exchange, which is critical for bone regeneration. Similarly, β-TCP is osteoconductive, providing a scaffold for bone cells and gradually resorbing to be replaced by new bone [43]. This material stimulates bone formation and enhances the osteogenic activity of stem cells, making it suitable for various bone regeneration applications. In conclusion, the materials used for EV transport and bone regeneration, including biodegradable polymers, scaffolds, hydrogels, collagen sponges, and β-TCP, each possess specific characteristics that enhance the delivery and effectiveness of EVs [43]. These materials address challenges such as retention at defect sites and sustained therapeutic action, contributing to improved outcomes in bone healing and regeneration. The ongoing development and optimization of these materials will be crucial for advancing regenerative medicine strategies [41,42,43] (Table 2).

## 6. Challenges and Perspectives for Clinical Applications

The clinical application of MSC-EVs in bone regeneration presents several challenges, particularly in terms of formulation, preservation, and administration methods. Current manufacturing methods often rely on planar culture systems and fetal bovine serum, which may lack scalability and fail to meet the safety and efficacy standards required for clinical use [44]. Additionally, MSC-EV isolation methods, such as ultracentrifugation and filtration, can result in low-purity products that complicate dosage and consistency in therapeutic applications [5]. Many studies do not adhere to the International Society for Cellular Therapy (ISCT) standards for MSC characterization, leading to heterogeneity in MSC sources and a lack of standardized characterization protocols, further complicating the translation of preclinical findings to clinical settings [5,45]. Shuji Terai et al. [7] indicate that intravenous administration of MSCs in clinical settings requires meticulous attention due to their larger size, which can lead to entrapment in capillaries, particularly in the lungs, causing serious complications like embolic events. For these reasons, careful monitoring and preparation to manage risks are needed to ensure patient safety. Post-administration monitoring is also critical to address any delayed complications, highlighting the need for comprehensive protocols and institutional readiness [7].

Yu Li et al. have pointed out that the design of delivery systems must be carefully evaluated to avoid potential side effects and ensure effective pharmacokinetics [46]. The main application of modified EVs, in which specific drugs are encapsulated in EVs, is for Drug delivery systems (DDS), because EVs can deliver functional molecules to remote organs and specific tissues and cells. EVs, mainly nano-sized EVs such as exosomes, are reported to have lower immunogenicity and mutagenicity than viral carriers, and to be biocompatible. EVs have also been reported to be resistant to nucleic acid-degrading enzymes in blood. EVs have also been suggested to possess delivery-oriented properties, and it is anticipated that a delivery system with low toxicity, high stability, and strong specificity can be developed. EVs with the above features are being developed, and their therapeutic effects are being verified.

Recent engineering advancements in MSC-EVs have significantly improved their therapeutic efficacy for bone regeneration and addressing bone loss. A key development is the establishment of a scalable manufacturing platform utilizing stirred-tank reactors (STR) combined with microcarriers, which enhances cell density and promotes higher EV secretion compared to traditional static culture methods [44]. This system allows for continuous collection of EV-enriched conditioned media under serum- and xenogeneic-free conditions, ensuring high purity and clinically relevant yields of MSC-EVs [44]. Additionally, bioengineering techniques enable the loading of osteoinductive molecules into MSC-EVs, enhancing their ability to promote bone repair and regeneration. These innovations not only address the limitations of low yield and purity in native EV treatments but also position MSC-EVs as effective drug delivery systems for targeted therapies in bone-related conditions [44].

Despite these challenges, the prospects of MSC-EVs in bone regeneration are promising. A search of PubMed and ClinicalTrials.gov revealed no registered clinical trials specifically evaluating mesenchymal stem cell-derived extracellular vesicles (MSC-EVs) for bone regeneration. Notably, a recent study reported the use of lyophilized MSC apoptotic vesicles (MSC-apoVs) to enhance hemostasis and bone regeneration in trauma patients following third molar extraction [47]. These findings indicate emerging translational potential, although clinical validation remains limited. Recent advances in formulation technologies, such as the use of hydrogels and scaffolds, have shown potential for enhancing the therapeutic effects of MSC-EVs in bone tissue engineering [48]. Numerous preclinical studies have demonstrated their ability to promote osteogenesis and angiogenesis [48]. Recent clinical trials have begun to explore the efficacy of MSC-EVs in treating bone-related conditions, particularly periodontitis and other bone-related diseases [5]. Dongxue Wang et al. reported significant potential in enhancing local bone regeneration and improving patient outcomes by combining MSC-EVs with advanced biomaterials such as biodegradable hydrogels [48]. Continued research on the specific mechanisms of action of MSC-EVs, along with the optimization of their formulation and delivery methods, is crucial for translating these therapies into clinical practice [46,48].

## 7. Pro-Angiogenic and Immunomodulatory Properties

There are limitations of MSC-EV therapy of bone defect repair following tumor resection, which is the theoretical risk that these vesicles may inadvertently promote tumor aggressiveness, especially in cases of residual microscopic disease.

Adriana Bajetto et al. [49] have shown that ADSC-EVs promoted glioma cell growth by stimulating the cell cycle, whereas, in contrast, BMSC-EVs and HucMSC-EVs inhibited glioma cell growth and increased apoptosis. This means that the tissue source of MSCs may have different effects on tumor behavior [49]. Virinder Kaur Sarhadi et al. [39] reported that ADSC-EVs have been shown to promote osteosarcoma (OS) cell growth, invasion, and migration. These EVs can increase the expression of COLGALT2 in OS cells, enhancing their invasive capabilities. Additionally, ADSC-EVs modulate the tumor microenvironment by influencing the expression of matrix metalloproteinases and other factors that facilitate bone remodeling, creating a supportive niche for tumor growth. ADSC-EVs engage in bidirectional communication with OS cells, leading to a reciprocal enhancement in tumor aggressiveness. This crosstalk involves the transfer of microRNAs and proteins that promote tumor cell migration and survival. Furthermore, ADSC-EVs contribute to the formation of a premetastatic niche by carrying factors that prepare distant sites for tumor cell colonization, thereby facilitating metastasis. Through these mechanisms, ADSC-EVs play a significant role in tumor progression, making them potential targets for osteosarcoma treatment [39].

They also report that BMSC-EVs promote OS cell proliferation, migration, and invasion by activating oncogenic pathways. The lncRNA MALAT1 in these EVs influences the miR-143/NRSN2/Wnt/β-catenin signaling axis, enhancing tumor growth and aggressiveness. BMSC-EVs also support oncogenic autophagy in OS cells, aiding their survival under stress conditions. Interactions between OS cells and BMSC-EVs can cause epigenetic changes, such as hypomethylation of LINE1 in MSCs, leading to a tumor-supportive phenotype. Additionally, BMSC-EVs enhance angiogenesis by transferring vascular endothelial growth factor-A (VEGF)-A to OS cells. These mechanisms highlight the role of BMSC-EVs in tumor progression, making them potential targets for osteosarcoma treatment [39].

Extracellular vesicles have emerged as important players in malignant tumor progression, particularly through their role in intercellular signaling within TME. These vesicles, derived from various cell types including MSCs and tumor cells, can transmit bioactive molecules such as proteins, lipids, and nucleic acids (including microRNAs) that influence receptor cell behavior, thereby promoting tumor progression [49].

Given the pro-angiogenic, immunomodulatory, and cell-proliferative effects of MSC-EVs, their use in oncologic settings demands thorough preclinical safety evaluations and careful patient selection. We have summarized the advantages, limitations, and potential oncological risks of each type of extracellular vesicle (EV) in Table 3.

## 8. Conclusions and Future Direction

In bone regeneration, MSC-EVs are considered promising therapeutic agents for repairing bone defects due to their ability to transport bioactive molecules, such as microRNAs and proteins, that promote osteogenesis and angiogenesis. Regenerative approaches utilizing MSC-EVs aim to achieve fundamental biological healing, with the potential to significantly improve the quality of life for patients with large or complex bone defects.

However, several limitations and challenges must be addressed before MSC-EVs can be widely adopted in clinical practice. Technically, issues related to large-scale production, formulation, and delivery methods remain unresolved. Furthermore, ethical and regulatory considerations require the development of standardized protocols to ensure safety, efficacy, and reproducibility. Importantly, there is a theoretical concern that MSC-EVs—due to their pro-angiogenic and immunomodulatory properties—may inadvertently promote tumor aggressiveness, particularly in cases of residual or microscopic malignancies following surgical resection of malignant bone or soft tissue tumors. This potential risk necessitates rigorous preclinical safety studies and careful patient selection in future clinical trials.

Moving forward, it will be essential to conduct well-designed clinical trials to evaluate the safety and therapeutic efficacy of MSC-EVs in humans. A multidisciplinary approach involving clinicians, bioengineers, and regulatory experts will be key to translating these advances into viable, safe, and effective therapies for patients suffering from challenging bone defects.

## Figures and Tables

**Table 2:** Materials and Their type, Characteristics for EV Transport and Bone Regeneration.

Material Type	(Sub Type)	Characteristics	Ref.
Biomaterial Scaffolds	Ceramic Scaffolds	– High biocompatibility and bioactivity – Supports bone mineralization – Often porous, allowing for cell infiltration and nutrient exchange	[42]
	Polymer Scaffolds	– Flexible and can be tailored for specific mechanical properties – Can be engineered for controlled degradation rates – Often used for their ability to be modified for enhanced EV loading and release	[42]
	Composite Scaffolds	– Combine properties of both ceramics and polymers – Enhanced mechanical strength and bioactivity – Can be designed to optimize EV delivery and retention	[42]
Three-Dimensional Tissue Engineering Scaffolds	-	– Support for Cell Growth: – Integration with Delivery Systems – Facilitation of Bone Healing	[41]
Hydrogels	-	– High Water Content – Flexibility – Enhanced Cell Communication – Biocompatible and can be modified to improve cell adhesion and growth	[42]
Electrospun Nanofibers	-	– Mimic the extracellular matrix structure, promoting cell attachment and growth – Can be designed to have specific surface properties for enhanced EV loading and release – Allow for the creation of a reservoir layer that can control the release of EVs	[42]
Bioactive Coatings	-	– Enhance the interaction between EVs and scaffolds – Can include adhesion molecules (e.g., fibronectin) to improve EV retention – Help in promoting osteogenic activity and tissue integration	[42]
Cationic Polymers	-	– Charge Reversal – Esterase-Responsive – Improved Uptake Efficiency	[43]
Collagen Sponge	-	– Natural Biomaterial – Porosity – Support for Cell Growth	[43]
β-TCP	-	– Osteoconductive – Biodegradable – Stimulates Bone Formation	[43]
Biodegradable Polymers	PLGA PEG	– Controlled Release – Biocompatibility – Degradability	[41]

EV, extracellular vesicle; β-TCP, Beta-tricalcium phosphate; PLGA, Poly lactic-co-glycolic acid; PEG, Polyethylene glycol.

**Table 3:** Summary of each EV’s insight.

EV Origin	Advantages	Limitations	Potential Oncological Risks	Refs.
ADSC-EVs	Therapeutic Potential: Rich in bioactive molecules (proteins, lipids, RNAs, and microRNAs) that regulate inflammation, apoptosis, and tissue regeneration. Promote Healing: Enhance angiogenesis, cell survival, and accelerate wound healing, particularly in diabetic and osteoporotic models. Mechanisms of Action: Activate critical signaling pathways (e.g., PI3K/Akt and STAT3) essential for cell proliferation, migration, and differentiation.	Source Variability: The effects of ADSC-EVs can vary based on the tissue source of the MSCs, which may lead to inconsistent therapeutic outcomes.	Tumor Promotion: ADSC-EVs may promote glioma cell growth by enhancing the cell cycle and have been shown to increase OS cell growth, invasion, and migration, indicating a potential role in tumor progression.	[8,9,10,11,39,49]
BMSC-EVs	Bone Regeneration: Significant potential in promoting bone regeneration and treating osteoporosis. Bioactive Content: Contain various bioactive molecules that facilitate intercellular communication and influence recipient cell behavior. Mechanistic Insights: Activate key signaling pathways (e.g., Wnt/β-catenin, BMP-2/Smad1/RUNX2) crucial for osteoblast differentiation and function.	Oncogenic Effects: BMSC-EVs can promote OS cell proliferation and migration through oncogenic pathways, indicating a dual role in both regeneration and tumor support.	Tumor Support: BMSC-EVs may enhance tumor growth and aggressiveness by transferring oncogenic factors and supporting autophagy in OS cells.	[14,15,39]
DPSC-EVs	Osteogenic Potential: Promote osteogenic differentiation in MSCs and enhance the expression of osteogenic markers. Mechanistic Action: Activate the MAPK signaling pathway, particularly through ERK and JNK pathways, which are crucial for osteogenic effects. In Vivo Efficacy: Enhanced new bone formation in critical-sized defect models treated with DPSC-EVs.	Research Focus: Future research is needed to optimize therapeutic applications and identify specific proteins and microRNAs that contribute to their regenerative properties.	Limited Information: No explicit mention of oncological risks associated with DPSC-EVs, but caution is warranted given the potential for any stem cell-derived product to influence tumor behavior.	[3,19,20,49]
HucMSC-EVs	Biocompatibility: High biocompatibility and low immunogenicity, making them suitable for therapeutic applications. Modulatory Effects: Enhance osteogenic differentiation and promote angiogenesis through critical signaling pathways (Wnt and Hippo). Immunomodulation: Polarize macrophages towards an anti-inflammatory phenotype, aiding tissue repair.	Optimization Needs: Current research trends focus on optimizing delivery systems and biomaterials to enhance bioavailability and efficacy.	Limited Evidence: No specific oncological risks for HucMSC-EVs, but their role in modulating immune responses could have implications for tumor interactions.	[22,23,49]

## Abbreviations

The following abbreviations are used in this manuscript:

TLA	Three-letter acronyms
MSC-EVs	Mesenchymal stromal cell-derived extracellular vesicles
ADL	Activities of daily living
mRNAs	Messenger RNAs
BMPs	Bone morphogenetic proteins
TME	Tumor microenvironment
MSCs	Mesenchymal stromal cells
EVs	Extracellular vesicles
ADSC-EVs	Adipose-derived stem cell extracellular vesicles
BMSC-EVs	Bone marrow-derived mesenchymal stromal cell extracellular vesicles
USP7	Ubiquitin-specific peptidase 7
DPSC-EVs	Dental pulp stem cell-derived extracellular vesicles
ALP	Alkaline phosphatase
OCN	Osteocalcin
HucMSC-EVs	Human umbilical cord mesenchymal stem cell-derived extracellular vesicles
MABs	Mesoangioblasts
IdU	Idoxuridine
Mu-EVs	Skeletal muscle secretes extracellular vesicles
BMSCs	Bone marrow mesenchymal stem/stromal cells
miR-208a	MicroRNA-208a
SMURF1	Smad ubiquitination regulatory factor 1
SPRY2	Sprouty2
COL-I	Type I collagen
OPN	Osteopontin
Sufu	Suppressor of fused homolog
ANG-1	Angiopoietin-1
PLGA	Poly lactic-co-glycolic acid
PEG	Polyethylene glycol
(ISCT	International Society for Cellular Therapy
DDS	Drug delivery systems
OS	Osteosarcoma
VEGF	Vascular endothelial growth factor
